# Severe maternal morbidity following stillbirth in Western Australia 2000–2015: a population-based study

**DOI:** 10.1007/s00404-022-06782-z

**Published:** 2022-09-15

**Authors:** Helen D. Bailey, Akilew A. Adane, Scott W. White, Brad M. Farrant, Carrington C. J. Shepherd

**Affiliations:** 1 Curtin Medical School, Faculty of Health Sciences, Curtin University, Perth, GPO Box U1987, 6845 Australia; 2 Telethon Kids Institute, The University of Western Australia, West Perth 6872, P.O. Box 855, Nedlands, WA Australia; 3 Ngangk Yira Institute for Change, Murdoch University, Murdoch, WA Australia; 4 Division of Obstetrics and Gynaecology, The University of Western Australia, Nedlands, WA Australia; 5 Maternal Fetal Medicine Service, King Edward Memorial Hospital, Subiaco, WA Australia

**Keywords:** Stillbirth, Obstetric complications, Severe acute maternal morbidity, Western Australia

## Abstract

**Purpose:**

There is scant literature about the management of stillbirth and the subsequent risk of severe maternal morbidity (SMM). We aimed to assess the risk of SMM associated with stillbirths compared with live births and whether this differed by the presence of maternal comorbidities.

**Methods:**

In this retrospective cohort study, we used a population-based dataset of all stillbirths and live births ≥ 20 weeks’ gestation in Western Australia between 2000 and 2015. SMM was identified using a published Australian composite for use with routinely collected hospital morbidity data. Maternal comorbidities were identified in the Hospital Morbidity Data Collection or the Midwives Notification System using a modified Australian chronic disease composite. Multivariable Poisson regression was used to estimate relative risks (RRs) and 95% confidence intervals (CIs) for factors associated with SMM in analyses stratified by the presence of maternal comorbidities. Singleton and multiple pregnancies were examined separately.

**Results:**

This study included 458,639 singleton births (2319 stillbirths and 456,320 live births). The adjusted RRs for SMM among stillbirths were 2.30 (95% CI 1.77, 3.00) for those without comorbidities and 4.80 (95% CI 4.11, 5.59) (Interaction *P* value < 0.0001) for those with comorbidities compared to live births without and with comorbidities, respectively.

**Conclusion:**

In Western Australia between 2000 and 2015, mothers of stillbirths both with and without any maternal comorbidities had an increased risk of SMM compared with live births. Further investigation into why women who have had a stillbirth without any existing conditions or pregnancy complications develop SMM is warranted.

**Supplementary Information:**

The online version contains supplementary material available at 10.1007/s00404-022-06782-z.

## What does this study add to the clinical work

**Table:** 

Women with and without comorbidities who had a stillbirth had a higher risk of severe maternal morbidity than those who had a livebirth, highlighting the need for careful monitoring around the birth.	

## Background

Stillbirth remains a global public health issue [1], with far reaching consequences for the wellbeing of parents, families and communities. This extends to impacts on wellbeing, relationships, functioning, and social and economic costs. The effects on mental health are frequently discussed in the literature [2], while little attention has been given to adverse physical health outcomes among mothers following a stillbirth despite adverse maternal outcomes [3–5] sharing certain risk factors for stillbirth—such as primiparity, maternal age over 35 years, pre-existing conditions such as diabetes, placental disorders, hypertensive disorders during pregnancy and low socio-economic status [6–10].

Three US studies [8–10] have found associations between stillbirth and more severe maternal morbidity. Gold et al. ([10]) found higher rates of some birth complications (including retained placenta and postpartum haemorrhage) among women with a stillbirth in Michigan compared to national prevalence estimates among live births [10] and Wall-Weiler et al. ([9]) a nearly fivefold higher risk using a composite indicator of severe maternal morbidity (SMM) among women with a stillbirth in California [9]. Finally, Lewkowitz et al. ([8]) reported a sixfold increased risk of SMM among women with existing comorbidities and, of more concern, a sevenfold increase among women without any existing comorbidities in Florida [8]. They used a modified, unweighted version of an obstetric comorbidity index developed by Bateman [4] to identify comorbidities in the birth hospitalisation (International Classification of Diseases (ICD), 9^th^ Revision, Clinical Modification (ICD-9-CM) diagnosis codes). Items included cardio-vascular conditions, chronic respiratory or renal disease and preeclampsia. A notable omission to this comorbidity index was placental abruption and placenta accreta, which has now been included in an expanded version of the comorbidity index using ICD-10-CM [11]. The purpose of obstetric comorbidity indices is to predict SMM [4, 11] but many of the included items are also strongly associated with stillbirth—such as chronic conditions (e.g. pre-existing diabetes, hypertension, obesity [7], systemic lupus erythematosus, chronic renal and thyroid conditions [12]) and pregnancy complications (e.g. gestational hypertension, preeclampsia, placental abruption [7], and placenta accreta [11, 13]). The approach used by Lewkowitz et al. ([8]) underscores the importance of producing results that are stratified by comorbidity status in order to quantify the differential risk of SMM among women with and without existing comorbidities [8].

The findings about stillbirth and SMM have major implications for women and their care providers as current guidelines for care following a stillbirth [6, 14] make limited mention of maternal morbidity, such as uterine rupture, and only in relation to induction of labour, particularly following a previous caesarean section. A fuller investigation in other population groups is likely to support further consideration of the antenatal and labour management of women following a foetal demise. Accordingly, the primary aim of this study was to assess the risk of SMM among Western Australian mothers who have given birth to a stillborn baby compared with those of live births, with consideration of existing comorbidities. Additionally, we sought to investigate the following: (1) whether the risk of SMM varied by gestational age, mode of birth, and types of stillbirth (antepartum or intrapartum); (2) the most common conditions within the SMM composite indicator; and (3) the associations between individual comorbidity items and SMM among mothers of stillbirths.

## Methods

### Study population

**Fig. 1:**
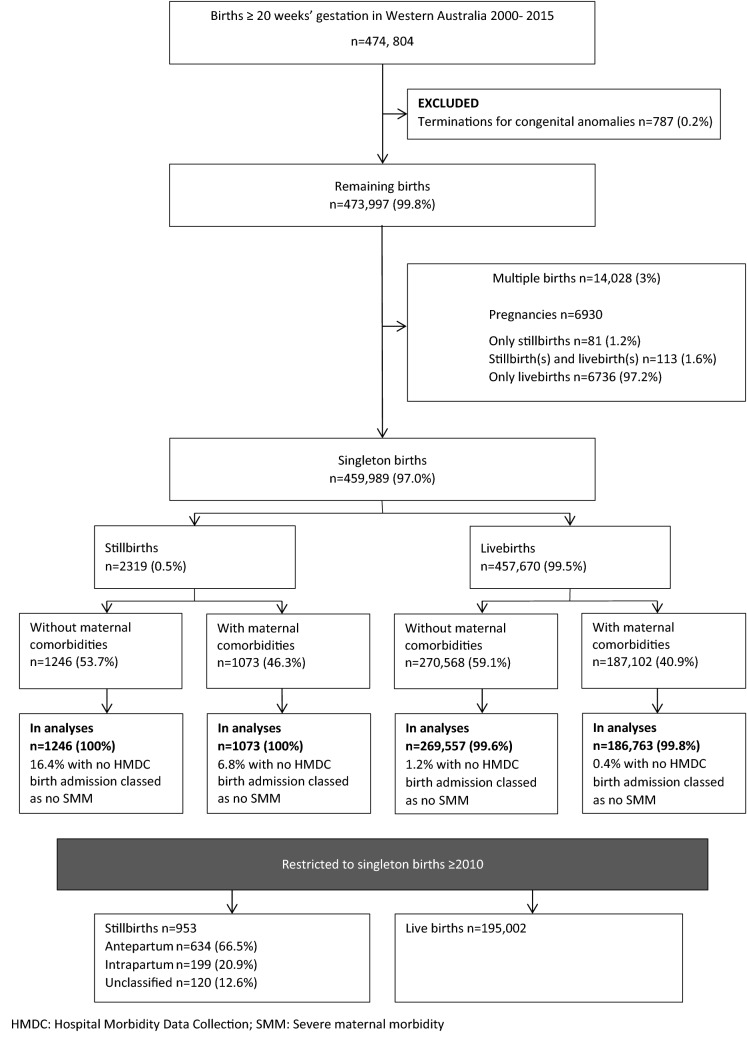
Study flow chart for investigation of Severe maternal morbidity following stillbirths in Western Australia 2000–2015

This study included all stillbirths and live births in Western Australia between 2000 and 2015. Separate cohorts were created for singletons and multiple births. Singleton births were stratified into subgroups with and without any maternal comorbidities, to replicate the approach used by Lewkowitz et al. [8] (who reported differences between these subgroups). Multiple births were examined at the pregnancy level, divided into three possible outcomes (only stillbirths, only livebirths and combination of both), although small sample sizes precluded an investigation by maternal comorbidities. (Fig. 1). For the investigation by type of stillbirth, the cohort consisted of all singleton births from 2010 to 2015 with stillbirth further categorised by the attending midwife as antepartum, intrapartum and unclassified, based on the timing of the fetal demise. This period coincides with the availability of quality data on stillbirth type—which was progressively introduced in Western Australian hospitals from 2005 but only available for nearly 90% of stillbirths from 2010.

### Data sources

This study used data obtained from population health datasets, mainly those which are routinely linked, using probabilistic matching techniques [15], by the Data Linkage team of the Western Australian Department of Health with identifying fields removed. The base dataset was the Midwives Notification System (MNS) which contains information about all live births and stillbirths from 20 weeks’ gestation or with a birthweight of 400 g or more in Western Australia. Other datasets included the Hospital Morbidity Data Collection (HMDC), which has data about all inpatient episodes in all Western Australian hospitals (public and private), Birth and Death Registers, and the Western Australian Register of Developmental Anomalies which contains information based on statutory notification of congenital anomalies diagnosed, including those leading to pregnancy termination.

### Data management

Stillbirths were categorised by attending midwives as antepartum, intrapartum and stillbirths based on when foetal demise was known to have occurred, and unclassified if this could not be determined.

### Pregnancy and birth related covariates

Maternal age at the birth was categorised into four groups (< 25, 25–29, 30–34 and > 34 years) while maternal self-reported ethnic origin categorised as Caucasian, Aboriginal and/or Torres Strait Islander (hereafter respectfully referred to as Aboriginal) and Other, based on the 8-part MNS classification (Caucasian, Aboriginal, Asian, Indian, African, Polynesian, Maori and Other). Socio-economic status (SES) was categorised using tertiles of an area-based measure of the birth residence [16] based on the Western Australian distribution of small area (mean population 400) values of the Index of Relative Socio-Economic Disadvantage, generated for the Census closest to the birth year. Areas of remoteness of birth residence (based on categories of the Accessibility/Remoteness Index of Australia [17]: very remote/remote and major cities/inner regional/outer regional) and any smoking during pregnancy (yes/no) were dichotomised.

Gestational age, based on ultrasound dating prior to (71%) or from 20 weeks (2%) or clinical signs/last menstrual period dates (24%) was categorised into five groups (20–23, 24–27, 28–31, 32–36 and ≥ 37 weeks). Parity was categorised into three groups (0, 1, ≥ 2). Onset of labour was categorised as spontaneous, induced or no labour (prelabour caesarean section) and mode of birth as vaginal or caesarean section. Among the multiple pregnancies, plurality was categorised into two groups (2, ≥ 3).

### Maternal comorbidities composite

We identified maternal comorbidities by adapting a chronic disease composite developed in Australia which used the ICD, Tenth Revision, Australian Modification (ICD-10-AM) diagnostic codes [18] with the inclusion of additional diagnoses from the US obstetric comorbidity index [4] modifications by Lewkowitz et al. [8] and Leonard et al. [11]. The latter of these expanded the index to include items such as placental abruption, which is a risk factor for stillbirth [19], and pre-existing bleeding disorders. These codes were converted into ICD-9-AM (for in-scope discharges) and ICD-10-AM, and modifications were made to reflect coding changes with the assistance of the Western Australian Clinical Coding Authority. Thus, the final comorbidity composite used in this study consisted of two parts: chronic and pregnancy-specific conditions (Table S1). Women were classified as having a chronic condition if any were noted in the MNS (pre-existing diabetes, essential hypertension, asthma and obesity (pre-pregnancy body mass index of ≥ 30 (Kg/m2), only from 2012) or were listed as a principal or additional diagnosis in the HMDC in any hospital separation from one year prior to conception until the index birth. Women were classified as having a pregnancy-specific condition (gestational diabetes or hypertension, pre-eclampsia, placenta praevia, placenta accreta, placental abruption) if noted in the MNS or were listed as a principal or additional diagnosis in the HMDC during any pregnancy admission. The final pregnancy related component was prior caesarean section, which was determined from the MNS data about the index birth and the mother’s previous births.

### Outcomes

The outcome of interest was SMM, which was sourced from the HMDC birth admission record, including subsequent inter-hospital transfers and Death Register data. SMM was identified using a published composite for use with routinely collected hospital morbidity data developed in Australia which used ICD-10-AM diagnostic and Australian Classification of Health Interventions (ACHI) procedure codes [20] (Table S2), which was updated to reflect coding changes. Other versions of the SMM composite were also made, by excluding the two most prevalent items, transfusion and curettage in combination with a general anaesthetic (hereafter curettage), one at a time and both together to investigate the impact of rarer SMM items. There is the potential for the diagnoses in the SMM composite to be the underlying cause of the stillbirth (e.g., uterine rupture), but we did not have information about the sequence or timing of events. Therefore, SMM was categorised into SMM based on any diagnosis (when SMM could have preceded the stillbirth) and SMM only based on procedures (which were more likely to reflect stillbirth and its management).

### Statistical analyses

The main analyses on singleton births were stratified by the presence of maternal comorbidities. Descriptive tables of general characteristics of stillbirths and live births were produced to identify potential co-variates. Poisson regression with cluster-robust standard errors (accounting for more than one birth event per woman during the study period) were used to estimate relative risks (RRs) and 95% confidence intervals (CIs) to investigate risk of SMM among women with stillbirths compared to those with live births, stratified by presence of comorbidity. Co-variates which changed the estimate by five percent or more were included in the multivariable model (only maternal ethnic origin). Further models were run using the versions of SMM excluding transfusion and/or curettage and individual components with at least 10 women in the stillbirth group. To assess whether results for those with and without maternal comorbidities were different, unstratified models with an interaction term were run. Models were then re-run, stratified by birth mode.

Further analyses were also performed, stratified only by gestational age groups, and timing of foetal demise (antepartum or intrapartum) as the numbers were too small to additionally stratify by maternal comorbidities.

Finally, among women with stillbirths only, models were run to investigate the association between SMM and maternal comorbidity and the individual components with at least 10 cases. These models were adjusted for parity group and mode of birth.

Analyses for multiple pregnancies were run comparing both those resulting in only stillbirth and stillbirth(s) and livebirth(s) to only livebirths. Because the initial results were similar for both groups, the stillbirth groups were combined into an ‘any stillbirth’ group.

### Missing data

Not all births (11.9% of stillbirths and 1.2% of live births) in the MNS had a HMDC birth admission record. For the majority, the most likely explanation is that there was no formal hospital admission as only short-term care was required. This is supported by MNS data that showed that: (1) those without a birth admission rarely had a maternal chronic condition or pregnancy or birth risk factor/complication; (2) nearly always had a vaginal birth (all stillbirths and 97.1% of live births); and (3) stillbirths mainly occurred prior to 32 weeks’ gestation (63.8%) while live born babies were mostly discharged on the day of birth (74.5%) (Table S3). Therefore, all stillbirths and live births discharged home on the day of birth were categorised as not having SMM so as not to over-estimate the prevalence of SMM, particularly among the stillbirths.

### Sensitivity analyses

As mothers could have had more than one birth (up to 10) during the study period, the analyses were limited to one randomly chosen birth per mother to address potential clustering effects. To facilitate easier comparison of the SMM findings with the existing literature [8, 9], which based maternal comorbidity only on hospital admission diagnoses, we repeated the SMM analyses using a version of the maternal comorbidity composite which used only the HMDC for all items except prior caesarean section. Finally, because placental abruption was the individual maternal comorbidity component with highest risk of SMM during delivery of a stillbirth, we repeated the stillbirths and live births analyses excluding those with placental abruption.

Cell counts with less than 10 were not tabulated to ensure confidentiality.

All analyses were conducted using SAS version 9.4 (SAS Institute Inc, Cary, NC, USA).

## Results

Between 2000 and 2015, there were 474,804 births. Of these 458,639 were singleton births (99.7%) who met the inclusion criteria for the study and had data for SMM (2319 stillbirths, 0.5% and 456,320 live births, 99.5%) and 6,930 were multiple pregnancies (81 resulting in only stillbirths, 1.2%; 113 stillbirth(s) and livebirth(s), 1.6%; and only livebirths, 97.2%) (Fig. 1). Among the singleton births, there were 953 stillbirths (634 antepartum, 199 intrapartum and 119 unclassified) and 195,0002 live births restricted to births between 2010 and 2015.

### Singleton births

Mothers of 46.3% of stillbirths and 40.9% of live births had at least one condition in the maternal comorbidity composite. Regardless of the presence of comorbidities, stillbirths were more likely to be born at an earlier gestation than live births (Table 1). Their mothers were more likely to be aged under 25 or over 34 years, be Aboriginal, have smoked during pregnancy and live in area in the lowest SES tertile than mothers of live births, with the highest prevalence of all these factors among those with comorbidities (Table 1). Both stillbirths and live births to mothers with any comorbidity were more likely to be born by caesarean section, although the proportion among stillbirths was far lower than among live births. The proportion of women of a stillbirth with SMM was 9.0% overall, 4.3% in those without any comorbidity, and 14.4% in those with any comorbidity compared to 2.1%, 1.7% and 2.7%, respectively, among live births (Table 2). The adjusted RRs (aRR) for SMM among women delivering a stillbirth were 2.30 (95% CI 1.77, 3.00) among those without comorbidities and 4.80 (95% CI 4.11, 5.59) among those with comorbidities compared to live births without and with comorbidities, respectively (Table 2, Interaction P value < 0.001). The strength of the associations fluctuated slightly when transfusion (the most prevalent of the individual SMM procedure) and next most prevalent procedure (curettage) were excluded. Without both these procedures, there was no association with SMM among those without comorbidities. A higher proportion of SMM was only based on procedures than based on any diagnosis. The aRRs for SMM only based on procedures were similar to overall SMM while the aRRs for SMM based on any diagnoses were higher. There were also elevated aRRs for both transfusion (6.44, 95% CI 5.30, 7.82 and 1.73 95% CI 1.10, 2.70) and curettage (15.73, 95% CI 10.46, 23.64 and 11.48 95% CI 7.73, 17.05) among those with and without comorbidities respectively). Among those with comorbidities only, the aRRs for hysterectomy, ventilation and uterine rupture were elevated—although these estimates were imprecise. Apart from curettage, the RRs were higher among those with comorbidities than without. The estimates for SMM among women delivering a stillbirth vaginally were similar to the main findings, but higher for women with comorbidities who had a stillbirth by caesarean. There were few women without comorbidities who had a stillbirth by caesarean so no analyses were performed.

**Table 1:** Maternal and other factors associated with singleton stillbirth at 20 or more weeks’ gestation, stratified by maternal comorbidities^a^ in Western Australia (2000–2015)

	Without maternal comorbidities				With maternal comorbidities				
	Stillbirth		Livebirth		Stillbirth			Livebirth	
	*n* = 1246		*n* = 269,557		*n* = 1073			*n* = 186,763	
	*n*	*%*	*n*	*%*	*n*	%		*n*	*%*
Maternal age (years)									
< 25	333	*26.8*	60,044	*22.3*	233	*21.7*		31,563	*16.9*
25–29	328	*25.6*	79,805	*29.6*	258	*24.0*		48,863	*26.2*
30–34	353	*27.7*	84,709	*31.4*	314	*29.3*		61,558	*33.0*
> 34	254	*19.8*	44,999	*16.7*	268	*25.0*		44,779	*24.0*
Parity									
0	670	*53.8*	129,637	*48.1*	348	*32.4*		60,220	*32.2*
1	275	*22.1*	81,311	*30.2*	341	*31.8*		73,888	*39.6*
≥ 2	290	*20.3*	57,8679	*21.5*	383	*35.7*		52,441	*28.1*
Maternal ethnic origin									
Caucasian	823	*66.1*	209,871	*77.9*	719	*67.0*		146,716	*78.6*
Aboriginal	150	*12.0*	15,572	*5.8*	169	*15.8*		10,591	*5.7*
Other	262	*21.0*	43,384	*16.1*	184	*17.1*		29,242	*15.7*
SES area birth residence tertiles									
Lowest	517	*41.5*	91,313	*33.9*	495	*46.1*		64,326	*34.4*
Middle	371	*29.8*	86,186	*32.0*	293	*27.3*		59,783	*32.0*
Highest	297	*23.8*	81,330	*30.2*	254	*23.7*		56,140	*30.1*
Missing values^b^	61	*4.9*	10,728	*4.0*	31	*2.9*		6514	*3.5*
Remote or very remote birth residence	173	*13.9*	28,226	*10.5*	137	*12.8*		16,443	*8.8*
Smoker during pregnancy	242	*19.4*	40,206	*14.9*	282	*26.3*		27,556	*14.8*
Gestational age (completed weeks)									
20–23	446	*35.8*	135	*0.1*	347	*32.3*		119	*0.1*
24–27	155	*12.4*	471	*0.2*	146	*13.6*		645	*0.3*
28–31	119	*9.6*	897	*0.3*	137	*12.8*		1536	*0.8*
32–36	192	*15.4*	12,346	*4.6*	184	*17.1*		15,035	*8.1*
≥ 37	312	*25.5*	254,473	*94.4*	244	*22.7*		168,771	*90.4*
Onset of labour									
Spontaneous	439	*35.2*	170,561	*63.3*	336	*31.3*		63,280	*33.9*
Induced	764	*61.3*	76,758	*28.5*	638	*59.5*		53,438	*28.6*
Prelabour caesarean section	19	*1.5*	21,041	*7.8*	93	*8.7*		69,409	*37.2*
Birth method									
Vaginal	1177	*94.5*	218,610	*81.1*	922	*85.9*		89,163	*47.7*
Caesarean section	45	*3.6*	49,751	*18.5*	145	*13.5*		96,966	*51.9*

^a^Maternal comorbidities were chronic or pregnancy-specific conditions recorded in the Midwives Notification System or Hospital Morbidity Data Collection (see Table S1 for details)

^b^Missing values only given if > 3% missing

**Table 2:** The risk of severe maternal morbidity (SMM) during the birth of a singleton stillbirth compared to livebirths in Western Australia (2000–2015), stratified by maternal comorbidities and by birth mode

	Without maternal comorbidities^a^			With maternal comorbidities^a^			Interaction *P* value^c^
	Stillbirth *n* = 1246	Livebirth *n* = 269,557	Adjusted^b^ RR (95% CI)	Stillbirth *n* = 1073	Livebirth 186,763	Adjusted^b^ RR (95% CI)	
	*n* (%)	*n* (%)		*n* (%)	*n* (%)		
All births							
SMM composite	54 (4.3)	4653 (1.7)	2.30 (1.77, 3.00)	155 (14.4)	5010 (2.7)	4.80 (4.11, 5.59)	< 0.001
SMM composite without transfusion	40 (3.2)	2732 (1.0)	3.01 (2.21, 4.09)	76 (7.1)	2835 (1.5)	4.29 (3.42, 5.38)	0.066
SMM composite without curettage with general anaesthetic	31 (2.5)	4281 (1.6)	1.43 (1.01, 2.03)	129 (12.0)	4800 (2.6)	4.16 (3.50, 4.93)	< 0.001
SMM composite without transfusion and curettage with general anaesthetic	15 (1.2)	2281 (0.8)	1.35 (0.82, 2.24)	49 (4.6)	2580 (1.4)	3.03 (2.28, 4.03)	0.006
SMM composite based on any diagnosis	235 (0.1)	< 10	8.37 (4.15, 16.9)	607 (0.3)	28 (2.6)	7.98 (5.45, 11.66)	0.949
SMM composite based only on procedures	4418 (1.6)	46 (3.7)	2.06 (1.54, 2.74)	4403 (2.4)	127 (11.8)	4.44 (3.74, 5.27)	< 0.001
Individual procedures^d^							
Transfusion	19 (1.5)	2085 (0.8)	1.73 (1.10, 2.70)	105 (9.8)	2418 (1.3)	6.44 (5.30, 7.82)	< 0.001
Curettage with general anaesthetic	26 (2.1)	463 (0.2)	11.48 (7.73, 17.05)	27 (2.5)	262 (0.1)	15.73 (10.46, 23.64)	0.236
Hysterectomy	0	23 (0.0)		13 (1.2)	251 (0.1)	8.14 (4.54, 14.62)	
Ventilation	< 10	30 (0.0)		11 (1.0)	81 (0.0)	21.31 (11.27, 40.32)	
Individual diagnoses^c^							
Uterine rupture	< 10	51 (0.0)		10 (0.9)	235 (0.1)	7.06 (3.74, 13.34)	
Vaginal births							
SMM composite	49 (3.9)	3642 (1.4)	2.29 (1.74, 3.02)	114 (10.6)	2198 (1.2)	4.58 (3.82, 5.49)	< 0.001
SMM composite without transfusion	37 (3.0)	2247 (0.8)	2.89 (2.1, 3.99)	54 (5.0)	1274 (0.7)	3.85 (2.94, 5.04)	0.004
SMM composite without curettage with general anaesthetic	26 (2.1)	3281 (1.2)	1.34 (0.92, 1.97)	88 (8.2)	2004 (1.1)	3.85 (3.13, 4.74)	< 0.001
SMM composite without transfusion and curettage with general anaesthetic	12 (1.0)	1808 (0.7)	1.17 (0.66, 2.05)	27 (2.5)	1040 (0.6)	2.34 (1.6, 3.44)	0.002
SMM composite based on any diagnosis	< 10			14 (1.3)	135 (0.1)	9.96 (5.68, 17.48)	
SMM composite based only on procedures	44 (3.5)	3515 (1.3)		100 (9.3)	2063 (1.1)	4.27 (3.52, 5.19)	< 0.001
Individual procedures^d^							
Transfusion	17 (1.4)	1523 (0.6)	1.83 (1.14, 2.93)	76 (7.1)	1002 (0.5)	6.50 (5.16, 8.18)	< 0.001
Curettage with general anaesthetic	26 (2.1)	449 (0.2)	10.25 (6.91, 15.21)	27 (2.5)	237 (0.1)	10.32 (6.89, 15.45)	0.365
Caesarean section							
SMM composite	< 10	994 (2.0)		39 (26.9)	2791 (2.9)	8.46 (6.39, 11.19)	
SMM composite without transfusion	< 10	476 (1.0)		20 (13.8)	1552 (1.6)	8.16 (5.38, 12.38)	
SMM composite without curettage with general anaesthetic	< 10	984 (2.0)		39 (26.9)	2776 (2.9)	8.54 (6.45, 11.3)	
SMM composite without transfusion and curettage with general anaesthetic	< 10	466 (0.9)		20 (13.8)	1532 (1.6)	8.32 (5.5, 12.61)	
SMM composite based on any diagnosis	< 10	108 (0.2)		13 (9.0)	471 (0.5)	17.39 (10.22, 29.61)	
SMM composite based only on procedures	< 10	886 (1.8)		26 (17.9)	2320 (2.4)	6.71 (4.68, 9.61)	
Individual procedures^d^							
Transfusion	< 10	552 (1.1)		27 (18.6)	1402 (1.4)	11.07 (7.74, 15.83)	

*CI* confidence interval, *RR* relative risk, *SMM* severe maternal morbidity

^a^See Table S1 for full list of factors in the maternal comorbidities composite

^b^Adjusted for maternal ethnic origin

^c^The interaction *P* value was obtained by including an interaction term for stillbirth and maternal comorbidities in the model unstratified by maternal comorbidity

^d^See Table S2 for full list of factors in the severe maternal morbidity composite. Factors only tabulated and RRs estimated where at least 10 women in the stillbirth group had this item

For both stillbirths and live births, the rates of SMM were highest in the 20–23 weeks’ gestation groups (11.2% and 11.4% respectively). The pattern of rates for live births exhibited a consistent downward trend at subsequent gestational age groups, to 2% at 37 weeks or more. In contrast, the rates for stillbirths were 7–10% at each gestational age group after 20–23 weeks. (Figure S1, Table S4). Accordingly, the aRRs for SMM among stillbirths compared with live births by gestational age groups were highest in the ≥ 37 weeks’ gestation group (aRR 2.94, 95% CI 2.12, 4.08, Table S4). Similarly, the rates of curettage with general anaesthesia were highest in the 20–23 weeks’ gestation groups (4.9% and < 3.9%, respectively) but no aRRs were estimated because of small cell sizes. The rates of transfusion among stillbirths by gestation ranged from 4.5% to 7.2% with no clear trend while the rates among live births was highest at 20–23 weeks and declined to 0.9% among term births. The resulting aRRs for transfusion among stillbirths compared with live births by gestational age groups were highest in the term birth group (aRR 4.53, 95% CI 3.09, 6.65; Table S4). In the analyses restricted to births from 2010–2015, the aRRs for SMM following antepartum and intrapartum stillbirths compared to live births were similar (4.13, 95% CI 3.24, 5.25 and 4.42, 95% CI 2.93, 6.65 respectively) (Table 3).

**Table 3:** The risk of severe maternal morbidity (SMM) during the birth of a singleton stillbirth compared to livebirths in Western Australia (2010–2015), stratified by type of stillbirth

	Livebirth *n* = 194,483	Antepartum stillbirth *n* = 634		Intrapartum stillbirth *n* = 199		
	*n* (%)	*n* (%)	1. Compared to livebirths	*n* (%)	2. Compared to livebirths	3. Antepartum compared to intrapartum stillbirths
			Adjusted^a^ RR (95% CI)		Adjusted^a^ RR (95% CI)	Adjusted^a^ RR (95% CI)
All births						
SMM composite^b^	4204 (2.2)	62 (9.8)	4.13 (3.24, 5.25)	22 (11.1)	4.42 (2.93, 6.65)	0.93 (0.58, 1.50)
SMM composite without transfusion	2283 (1.2)	32 (5.0)	4.06 (2.89, 5.72)	15 (7.5)	5.89 (3.61, 9.60)	0.69 (0.38, 1.25)
SMM composite without curettage with general anaesthetic	3999 (2.1)	45 (7.1)	3.15 (2.36, 4.20)	14 (7.0)	2.94 (1.73, 4.99)	1.07 (0.59, 1.95)
SMM composite without transfusion and curettage with general anaesthetic	2045 (1.1)	15 (2.4)	2.14 (1.29, 3.54)	< 10		
Individual procedures^b^						
Transfusion	2084 (1.1)	38 (6.0)	4.91 (3.57, 6.76)	< 10		
Curettage with general anaesthetic	244 (0.1)	17 (2.7)	19.45 (11.96, 31.61)	< 10		
Vaginal births						
SMM composite^b^	2361 (1.9)	46 (7.9)	3.89 (2.93, 5.16)	19 (9.5)	4.82 (3.12, 7.47)	0.81 (0.48, 1.35)
Caesarean section						
SMM composite^b^	1808 (2.8)	16 (29.6)	10.01 (6.53, 15.35)	< 10		

*CI* confidence interval, *RR* relative risk, *SMM* severe maternal morbidity

^a^Adjusted for maternal ethnic origin

^b^See Table S2 for full list of factors in the severe maternal morbidity composite. for Individual factors only tabulated and RRs estimated where at least 10 women in the stillbirth group had this item

**Table 4:** The risk of severe maternal morbidity among women with a singleton stillbirth with and without maternal comorbidities, Western Australia, 2000–2015

Severe maternal morbidity^a^					
	With comorbidity		Without comorbidity		Adjusted RR (95% CI)^b^
	n	%	n^c^	%	
Any comorbidity^d^	155	14.4	54	4.3	2.65 (1.94, 3.63)
Any chronic/existing condition	60	12.2	149	8.2	1.33 (1.00, 1.76)
Cardio-vascular conditions	15	18.5	194	8.7	1.73 (1.07, 2.78)
Chronic respiratory disease/asthma	29	12.3	180	8.6	1.41 (0.98, 2.01)
Obesity	11	7.2	198	9.1	0.70 (0.38, 1.29)
Pregnancy complications/risk factors	139	17.4	70	4.6	3.03 (2.25, 4.10)
Placenta praevia	14	27.5	193	8.6	2.22 (1.35, 3.67)
Placental abruption	83	37.6	132	6.2	5.01 (3.89, 6.45)
Preeclampsia	22	15.8	185	8.6	1.86 (1.25, 2.75)
Previous caesarean section	54	13.6	154	8.1	0.98 (0.70, 1.38)

*CI* Confidence interval, *RR* Relative risk

^a^Of 2319 women with a singleton stillbirth, 2110 did not have any severe maternal morbidity

^b^Adjusted for birth mode and parity group

^c^This is the number and percentage of women without the specific factor, but who had severe maternal morbidity, using the total with the condition as the denominator

^d^See Table S1 for full list of factors. Individual factors only tabulated where the total number with factor was at least 10

Among only women with a stillbirth, the individual comorbidities most strongly associated with having SMM were placental abruption (aRR 5.01, 95% CI 3.89, 6.45), placenta praevia (aRR 2.22, 95% CI 1.35, 3.67), and preeclampsia (aRR 1.86, 95% CI 1.25, 2.75) (Table 4).

### Results of the sensitivity analyses

There were minimal changes to adjusted RRs for SMM among stillbirths when the analyses were restricted to one birth per mother (Table S5) or when only the HMDC was used to define comorbidity (except for prior caesarean section) (Table S6) despite the proportions of stillbirths and live births with any maternal comorbidities declining to 34.2% and 30.1%, respectively, in the latter analyses. When the analyses for SMM among those with any comorbidities were repeated excluding placental abruption, the aRR decreased to 3.04 (95% CI 2.43, 3.80) (Table S7).

### Multiple pregnancies

The proportion of multiple pregnancies resulting in any stillbirth that had at least one condition in the maternal comorbidity composite was 47.2% while it was 51.2% of pregnancies resulting in only livebirths. Like for singleton births, pregnancies resulting in any stillbirth were more likely to born at an earlier gestation than pregnancies resulting in only livebirths and their mothers were more likely to be Aboriginal with the remainder of characteristics being similar (Table S8). The proportion of stillbirth pregnancies with SMM was 8.8% compared to 3.8% among pregnancies resulting in only live births (Table S2). The aRR for SMM among pregnancies resulting in any stillbirth was 2.21 (95% CI 1.72, 2.82) (Table S9).

## Discussion

### Principal findings

We found that the rate of SMM in Western Australia between 2000 and 2015 was about 9% following a stillbirth compared to around 2% following a live birth. The elevated risk of SMM among women who had a stillbirth was doubled when a maternal comorbidity was present (aRR 4.8 vs 2.3 among those without a maternal comorbidity). Among the stillbirths, the maternal comorbidities most strongly associated with SMM were known risk factors for SMM—placental abruption [21] and placenta praevia [22].

### Strengths of the study

This population-based study adds to the limited information about the association between SMM and stillbirth. We used two data sources (MNS and HMDC) and a validated SMM composite [20] that was developed for application in Australian hospital administrative datasets, but which have also been used elsewhere [23, 24]. For maternal comorbidities, we adapted an accepted chronic disease composite [18]—adding other conditions and pregnancy complications from an expanded version [11] of the comorbidity index used by Lewkowtiz et al.[8]. Further, given our access to hospitalisations prior to the birth admission, we examined conditions in the year prior to conception since longer look-back periods can improve identification of chronic conditions [18]. Using the MNS data increased ascertainment of comorbidities and pregnancy complications that were underreported in the HMDC. For example, the prevalence of maternal chronic respiratory disease/asthma among stillbirths increased from 0.5% using HMDC alone to 9.5% by adding in the MNS data about asthma. In addition, the hospital electronic data collections which feed into the MNS [25] contain data entered during the pregnancy, so data entry about associated conditions may have predated the stillbirth occurrence, potentially reducing the risk of reporting bias. Thus, the combination of a broader definition of comorbidity, longer look-back period and additional data source may explain why we reported higher levels of comorbidity (46% of stillbirths and 41% of livebirths, respectively) than Lewkowitz et al. [8] (31% of both stillbirths and live births).

### Limitations of the data

A larger proportion of stillbirths than live births did not have a maternal hospital admission for the birth. As SMM is a rare event, treating these as missing would have led to an inflated SMM risk estimate among the stillbirths. Therefore, we took a cautious approach and assumed that short-term care was provided without an admission, and thus no SMM (which would have triggered an admission). This is supported by their low levels of MNS pregnancy complications and is consistent with evidence from a Swedish study that 9% of mothers of stillbirths had a hospital stay of < six hours (and > 40% 24 h or less) [26].

Routinely collected data collections do not include all information of interest, for example, cause of foetal death so we were unable to investigate the relationship of all relevant factors with SMM. We also did not have data about the chronology of events so cannot distinguish stillbirths which occurred prior (and contributed) to the SMM such as disseminated intravascular coagulation [27] from stillbirths that may have occurred directly as a consequence of the SMM, e.g. following uterine rupture [28], albeit these events were rare in our dataset.

### Interpretation

Our study adds to the scant literature on the increased risk of SMM associated with stillbirth in HICs [8, 9]. The first of two existing, large population-based studies used Californian birth data from 1999 to 2011 (~ 26,000 stillbirths) and reported a RR of 4.8 for SMM among mothers of stillbirths [9] while the second used birth data from Florida from 2005 to 2014 (~ 9,500 stillbirths) and reported RRs of 6.2 among women with a stillbirth with comorbidities and 7.0 without comorbidities compared to equivalent mothers of live births [8]. Like Wall-Weiler et al. [9] we found that the risk of SMM in stillbirths increased from about 28 to 29 weeks’ gestation compared to live births. Our finding of a higher risk of SMM among mothers of a stillbirth with any comorbidities by caesarean section is similar to Lewkowitz et al. [8] and is likely to reflect mainly the severity and urgency of the underlying problem such as placental abruption or placenta accreta, rather than the actual caesarean procedure.

Unlike previous studies [8–10], we distinguished between antepartum and intrapartum events but found similar levels of risk. However, the sample sizes for these analyses were small so the analyses need to be replicated in a larger study.

Both Lewkowitz et al. [8] and Wall-Weiler et al. [9] identified SMM in the birth admission using the CDC algorithm [29] which shared most of the items in the Australian SMM composite, [20], although the latter includes a wider range of diagnoses, which were included in an expanded version of the CDC composite [3]—such as uterine rupture, status asthmaticus and status epilepticus and procedures (notably curettage). Like us, they reported that mothers of stillbirths had an increased risk of transfusion (the most prevalent individual item) but the risk of SMM remained elevated when transfusion was excluded [8, 9]. However, both the Australian and CDC SMM composites share the limitation that the amount of transfusion was not quantified. Nevertheless, transfusion is a marker of severe haemorrhage so is consistent with placental abruption [9, 21] and placenta praevia [8, 9, 22] being predictors of SMM (as we found) and with there being a higher risk of postpartum haemorrhage among women with a stillbirth [10, 30]. Our finding that stillbirth is associated with an elevated risk of curettage (the second most common SMM item in our study which was not included in the CDC composite) is in keeping with an increased risk of retained placenta following stillbirth [10, 31, 32]. Other risk factors for retained placenta that make stillbirths more at-risk include early preterm birth [31, 33], preeclampsia and foetal growth restriction [34].

Current guidelines for care following stillbirth by the American College of Obstetricians and Gynecologists [6] and Royal College of Obstetricians and Gynaecologists [14] focus on the thorough investigation of the underlying cause of the stillbirth and supportive care for the parents rather than prescriptive details about labour management. This suggests that stillbirth is primarily a fetal problem and shows a lack of recognition of the important maternal morbidity that often accompanies stillbirth. When a pregnant woman has existing comorbidities such as pregnancy complications, clinicians can consult condition-specific guidelines on labour management, for example hypertension in pregnancy [35] and placenta accreta [36]. In the case of stillbirth without underlying morbidities, clinicians may benefit from more guidance about labour management and tools for counselling women. For example, current stillbirth guidelines by individual Australian States [37, 38] and hospitals [39] advocate offering active management of the third stage of labour. Notwithstanding, a 2013 Australian randomised controlled trial investigating different pregnancy termination regimes at 14–28 weeks gestation following foetal death reported that only around 64% of women had oxytocin in the third stage of labour [9]. Therefore, population-based estimates of curettage and transfusion may be useful when counselling patients about the need for active management [40].

## Conclusions

In Western Australia between 2000 and 2015, women with and without comorbidities who had a stillbirth had a higher risk of SMM. Among those without comorbidities, the elevated risk was due to transfusion and curettage in association with general anaesthetic. While an elevated risk of SMM among those with any comorbidities is not unexpected, given the shared antecedents for SMM [3–5] and stillbirth [6, 7], the finding about women without any existing conditions or pregnancy complications warrants further investigation. In turn, this may afford the opportunity for improved care guidelines.

## Supplementary Information

Below is the link to the electronic supplementary material.

Supplementary file1 (DOCX 218 KB)

## Data Availability

No additional data are available.

## Abbreviations

ACHI: Australian classification of health interventions
CI: Confidence interval
HMDC: Hospital morbidity data collection
ICD: International classification of diseases
ICD-10-AM: International classification of diseases, Australian modification
MNS: Midwives notification system
RR: Relative risk
SES: Socio-economic status
SMM: Severe maternal morbidity

## Code availability

The SAS code used to analyse the data can be obtained from the corresponding author upon reasonable request.
