# Supplementary material for: Severe maternal morbidity following stillbirth in Western Australia 2000–2015: a population-based study
Source: Arch Gynecol Obstet. 2022 Sep 15;308(4):1175–87. doi: 10.1007/s00404-022-06782-z (PMC10435652; doi:10.1007/s00404-022-06782-z)
Supplement: Supplementary file 1 — Supplementary file1 (DOCX 218 KB) [file 404_2022_6782_MOESM1_ESM.docx]

**Supplementary appendix:**

Authors: Helen D Bailey^1,2^ (ORCID 0000-0002-1259-3793), Akilew A Adane^2,3^ (ORCID 0000-0002-3022-5230)_,_ Scott W White^4,5^ (ORCID 0000-0002-7235-6407), Brad M Farrant^2^ (ORCID 0000-0002-3903-0990), Carrington CJ Shepherd^1-3^ (ORCID 0000-0003-0043-7053)

Affiliations:

^1^Curtin Medical School, Faculty of Health Sciences, Curtin University, Perth, Australia

^2^ Telethon Kids Institute, The University of Western Australia, Nedlands, Western Australia, Australia

^3^Ngangk Yira Institute for Change, Murdoch University, Murdoch, Western Australia, Australia.

^4^ Division of Obstetrics and Gynaecology, The University of Western Australia, Nedlands, WA, Australia

^5^Maternal Fetal Medicine Service, King Edward Memorial Hospital, Subiaco, Western Australia, Australia

**Corresponding Author**

Helen D Bailey

Curtin Medical School, Faculty of Health Sciences, Curtin University GPO Box U1987, Perth 6845, Western Australia, Australia.

Email: [Helen.bailey@curtin.edu.au](mailto:Helen.bailey@curtin.edu.au)

**Table of contents**

|  | Page number |
| --- | --- |
| Table S1: Conditions included as maternal comorbidities: International Classification of Disease (ICD) Australian Modification Versions 9 and 10 diagnostic code and diagnostic categories in the Hospital Morbidity Data Collection or Midwives Notification System (MNS) | 3 |
| Table S2. Components and ICD10 Codes of severe maternal morbidity (SMM) | 5 |
| Table S3. Comparison of maternal and other factors sourced from MNS among those with and without birth hospital admission data, stratified by birth status | 7 |
| Table S4. The risk of severe maternal morbidity (SMM) during the birth of a singleton stillbirth compared to livebirths in Western Australia (2000-2015), by gestational age group. | 8 |
| Table S5. The risk of severe maternal morbidity (SMM) during the birth of a singleton stillbirth compared to livebirths in Western Australia (2000-2015), stratified by maternal comorbidities, limited to one birth per mother. | 10 |
| Table S6. Severe maternal morbidity (SMM) among women during birth of a singleton stillbirth compared to livebirths in Western Australia (2000-2015), stratified by maternal comorbidities (based on HMDC data for everything except previous caesarean section) | 11 |
| Table S7. The risk of severe maternal morbidity (SMM) during the birth of a singleton stillbirth compared to livebirths in Western Australia (2000-2015), among those with maternal comorbidities, excluding placental abruption. | 12 |
| Table S8. Maternal and other factors associated with multiple pregnancies at 20 or more weeks’ gestation in Western Australia (2000- 2015). | 13 |
| Table S9 The risk of severe maternal morbidity (SMM) during the births of multiple pregnancies with any stillbirth compared to multiple pregnancies with only livebirths in Western Australia (2000-2015) | 15 |
| Figure S1: Rates of severe maternal morbidity by gestational age group among live births and stillbirths in Western Australia, 2000-2015. | 16 |

Table S1: Conditions included as maternal comorbidities ^a^: International Classification of Disease (ICD) Australian Modification Versions 9 and 10 diagnostic code and diagnostic categories in the Hospital Morbidity Data Collection or Midwives Notification System (MNS).

| Maternal conditions | ICD-9-AM codes | ICD-10-AM^b^ codes | | |
| --- | --- | --- | --- | --- |
|  |  | |  | Excluded if only in birth admission |
| Chronic/existing conditions^c^ |  | |  |  |
| Cardio-vascular conditions |  | |  |  |
| Acute/chronic rhematic fever | 390-398 | | I00-02, I05-09 |  |
| Hypertensive disorders | 401-405, 642.0-642.2 | | I10-15, O10 or recorded in MNS |  |
| Ischaemic heart diseases | 410-414 | | I20-25 | I21, I24 |
| Pulmonary heart diseases | 415-417 | | I26-28, O88 | I26, O88 |
| Other forms of heart disease | 420-429, 642.0- 642.2 | | I30-52, | I30, I33, I40, I46, I50 |
| Congenital anomalies | 648.5,745-746, 747.4 | | Q20-25, O99.4 |  |
|  |  | |  |  |
| Chronic renal conditions | 580-590, 593, 599.7, 753, 996.1, 996.81, V42.0, V45.1, V56.0, V56.8 | | N00-08, N11-12, N14-16, N18-19, N25-28, N39.1-N39.2, O26.81, Q60-63, T82.4, T86.1, Z49, Z94.0, Z99.2, | O26.81 |
| Chronic respiratory disease/asthma | 490-494, 496, 518.81 | | J40-47, J96.1, J96.9 or recorded in MNS |  |
| Pre-existing diabetes | 250 | | E10-11, E13-14, O24.0-24.3 or recorded in MNS |  |
| Thyroid disorders | 240-246 | | E00-07, E35.0, E89.0, Q89.22 |  |
| Auto immune conditions | 281.0, 287.3, 340, 358.0, 446, 447.6, 451, 555-556, 571.6, 671.3- 671.4, 673, 694.3, 695.4, 696, 709.1, 710.0, 710.3-710.4, 714, 720.0 | | D51, I77.6, I80, O22.3, O87.1, O88, G35, G70.0, K50-K51, K74.3, K75.4, K90.0, L40, L93, L95, M05, M06, M08.1, M45, M30-M33.2 | O22.3, O87.1 |
|  |  | |  |  |
| Cystic fibrosis | 277.0 | | E84 |  |
| Human immunodeficiency virus | 042, V08 | | B20, B24, O98.7, Z21 |  |
| Thalassaemia/sickle cell anaemia | 282.4, 282.6 | | D56-57 |  |
| Other diseases of the blood and blood forming organs | 286-287, 666.3 | | D65-D69, O87.1, O72.3 | D65, O72.3 |
| Obesity | 278.0-278.03 | | E66 |  |
| Pregnancy complications/risk factors |  | |  |  |
| Previous caesarean section |  | | If recorded in MNS |  |
| Gestational diabetes |  | | O24.4, O24.9 or recorded in MNS |  |
| Gestational hypertension |  | | O13, O16 |  |
| Preeclampsia |  | | O11, O14, O15 or recorded in MNS |  |
| Placenta Praevia |  | | O20.8, O20.9, O44-46 or recorded in MNS |  |
| Placental abruption |  | | O45 or recorded in MNS |  |
| Placenta accreta |  | | O43.2 |  |
|  |  | |  |  |

ICD: International Classification of Disease; MNS: Midwives Notification System

^a^. Based on the list of chronic diseases and pregnancy complications as defined by Chen 2011 [1], with additions from maternal medical comorbidity composite as used by Lewkowitz, 2019 [2] and Leonard [3]

^b^ Pregnancy conditions were only coded using ICD-10-AM codes as ICD-10-AM codes were introduced in July 1999.

^c^ Based on hospital admissions in the year prior to conception. Conditions only included in one condition group when overlap with another (e.g. E10.1 Type 1 diabetes with kidney complications only in Pre-existing diabetes group, and I15.1 Hypertension secondary to other kidney disorder only in Cardio-vascular condition group.)

1. Chen JS, Roberts CL, Simpson JM, Ford JB (2011) Use of hospitalisation history (lookback) to determine prevalence of chronic diseases: impact on modelling of risk factors for haemorrhage in pregnancy. BMC Med Res Methodol 11:68. doi:10.1186/1471-2288-11-68

2. Lewkowitz AK, Rosenbloom JI, Lopez JD, Keller M, Macones GA, Olsen MA et al. (2019) Association Between Stillbirth at 23 Weeks of Gestation or Greater and Severe Maternal Morbidity. Obstet Gynecol 134 (5):964-973. doi:10.1097/AOG.0000000000003528

3. Leonard SA, Kennedy CJ, Carmichael SL, Lyell DJ, Main EK (2020) An Expanded Obstetric Comorbidity Scoring System for Predicting Severe Maternal Morbidity. Obstet Gynecol 136 (3):440-449. doi:10.1097/AOG.0000000000004022

Table S2. Components and ICD10 codes of severe maternal morbidity (SMM)^a^

| Diagnoses | ICD-10-AM Diagnostic Codes | Changes in the current study |
| --- | --- | --- |
| Acute abdomen | K35, K37, K65.0, K65.9, N73.3, N73.5 | From 01/07/2010, K35.0/K35.1/K35.9 replaced by K35.2/K35.3/K35.8 |
| Acute renal failure | O90.4, N17, N19, N99.0 |  |
| Acute psychosis | F53.1, F23 |  |
| Cardiac arrest, failure or infarction | O89.1, O74.2, O90.3, I21, I42, I43, I46, I50, J81 | not J81 is assigned if, J81 is only used if a specified cause of the acute pulmonary oedema is documented. If the underlying cause is not documented, is used |
| Cerebral oedema or coma | G93.6, R40.2 |  |
| Disseminated intravascular coagulopathy | D65 |  |
| Cerebro-vascular accident | I60–I64 |  |
| Major complications of anaesthesia | O74.0, O74.2, O74.3, O89.0–O89.2, O29.0–O29.2 |  |
| Obstetric embolism | O88 |  |
| Shock | R57, O75.1, T80.5, T88.6 |  |
| Sickle cell anaemia with crisis | D57.0 |  |
| Status asthmaticus | J46 |  |
| Status epilepticus | G41 |  |
| Uterine rupture | O71.0, O71.1 | From 01/07/2008:  O71.0 replaced by O71.00, O71.01, O71.02  O71.1 replaced by O71.10, O71.11, O71.12 |
| Maternal death | (From Death Register) |  |
| Procedures | ACHI Procedure Codes |  |
| Assisted ventilation including tracheostomy | 13857-00, 13879-00, 13882-00, 13882-01, 13882-02, 92038-00, 92039-00, 92040-00, 92041-00, 41880-00, 41883-00, 41883-01, 90179-06, 92046-00, 92047-00 | From 01/07/2008:  13857-00, 13879-00 were replaced by 13882-00, 13882-01, 13882-02  92038-00, 92039-00, 92040-00 were replaced by 92209-00, 92209-01, 92209-02  41883-00, 41883-01 were replaced by 41881-00, 41881-01 |
| Curettage in combination with a general anaesthetic | 16564-00–01, 35640-00–03  92514-10–99, 92502-00–03 |  |
| Dialysis | 13100-00, 13112-00, 90351-00, 13109-00,13109-01, 13110-00 |  |
| Evacuation of haematoma | 90484-00, 90484-01, 90484-02, 30394-00 |  |
| Hysterectomy | 35653-00 to 03 | From 01/07/2008: 35653-02, 35653-03 replaced by 35653-04 |
| Procedures to reduce blood flow to uterus | 34103-12, 33833-03, 35321-00, 33845-00, 30385-00, 35759-00 | From 01/07/2008: 35321-00 replaced by 35321-06 |
| Reclosure of disrupted CS wound | 30403-03 |  |
| Repair of bladder or cystostomy | 90480-00, 37004-03, 37004-02, 37011-00, 37008-01 |  |
| Repair of intestine | 30566-00, 30375-19, 30565-00,  32069-00, 30375-24, 32003-00,  32000-00, 32003-01, 32000-01,  32005-01, 32004-01, 32006-00,  32006-01, 32005-00, 32004-00,  32012-00, 32009-00, 30375-25,  43816-02 |  |
| Repair ruptured or inverted uterus | 90478-00, 16570-01 | From 01/07/2008: 90478-00 removed |
| Transfusion of blood or  coagulation factors | 13706-01, 13706-02, 13706-03,  92061-00, 92062-00, 92206-00 |  |

^a^ From Roberts CL, Cameron CA, Bell JC, Algert CS, Morris JM. Measuring maternal morbidity in routinely collected health data: development and validation of a maternal morbidity outcome indicator. *Med Care.* 2008;46(8):786-794. Please see paper for more details.1. Chen JS, Roberts CL, Simpson JM, Ford JB (2011) Use of hospitalisation history (lookback) to determine prevalence of chronic diseases: impact on modelling of risk factors for haemorrhage in pregnancy. BMC Med Res Methodol 11:68. doi:10.1186/1471-2288-11-68

Table S3. Comparison of maternal and other factors sourced from MNS among those with and without birth hospital admission data, stratified by birth status.

|  | Stillbirth (n= 2319) | | | |  | Livebirth (n= 457,670) | | | | |
| --- | --- | --- | --- | --- | --- | --- | --- | --- | --- | --- |
|  | With data | | Without data | |  | With data | Without data | | |  |
| n (row%) | 2040 (88.0%) | | 279 (12.0%) | |  | 452,295 (98.8%) | | n=5375 (1.2%) | | |
|  | n | *%* | n | *%* |  | n | *%* | n | *%* |  |
| Maternal age (years) |  |  |  |  |  |  |  |  |  |  |
| <25 | 477 | *23.4* | 89 | *31.9* |  | 90,644 | *20.0* | 1310 | *24.4* |  |
| 25-29 | 504 | *24.7* | 73 | *26.2* |  | 127,479 | *28.2* | 1574 | *29.3* |  |
| 30-34 | 590 | *28.9* | 67 | *24.0* |  | 145,112 | *32.1* | 1543 | *28.7* |  |
| >34 | 469 | *23.0* | 49 | *17.6* |  | 89,060 | *19.7* | 948 | *17.6* |  |
|  |  |  |  |  |  |  |  |  |  |  |
| SES area birth residence tertiles |  |  |  |  |  |  |  |  |  |  |
| Lowest | 894 | *43.8* | 118 | *42.3* |  | 154,178 | *34.1* | 1935 | *36.0* |  |
| Middle | 579 | *28.4* | 85 | *30.5* |  | 144,684 | *32.0* | 1653 | *30.8* |  |
| Highest | 492 | *24.1* | 59 | *21.1* |  | 136,397 | *30.2* | 1428 | *26.6* |  |
| Missing values | 75 | *3.7* | 17 | *6.1* |  | 17,036 | *3.8* | 359 | *6.7* |  |
|  |  |  |  |  |  |  |  |  |  |  |
| Remote or very remote birth residence | 277 | *13.6* | 33 | *11.8* |  | 44,360 | *9.8* | 519 | *9.7* |  |
|  |  |  |  |  |  |  |  |  |  |  |
| Maternal ethnic origin |  |  |  |  |  |  |  |  |  |  |
| Caucasian | 1357 | *66.5* | 185 | *66.3* |  | 353,709 | *78.2* | 3898 | *72.5* |  |
| Aboriginal | 292 | *14.3* | 27 | *9.7* |  | 26,027 | *5.8* | 285 | *5.3* |  |
| Other | 383 | *18.8* | 63 | *22.6* |  | 71,616 | *15.8* | 113 | *21.1* |  |
|  |  |  |  |  |  |  |  |  |  |  |
| Birth method |  |  |  |  |  |  |  |  |  |  |
| Spontaneous vaginal | 1242 | *60.9* | 181 | *71.2* |  | 239,994 | *53.1* | 4730 | *88.0* |  |
| Instrumental vaginal | 634 | *31.1* |  |  |  | 142,590 | *31.5* | 519 | *9.7* |  |
| Caesarean section | 142 | *7.0* | <10 ^a^ | *<3.6* |  | 67,890 | *15.0* | 112 | *2.1* |  |
|  |  |  |  |  |  |  |  |  |  |  |
| Gestational age (weeks) |  |  |  |  |  |  |  |  |  |  |
| 20-31 | 1110 | *54.4* | 178 | *63.8* |  | 2923 | *0.6* | 61 | *1.1* |  |
| 32-36 | 402 | *19.7* | 36 | *12.9* |  | 28,226 | *6.2* | 220 | *4.1* |  |
| ≥37 | 497 | *24.4* | 59 | *21.1* |  | 419,263 | *92.7* | 4950 | *92.1* |  |
|  |  |  |  |  |  |  |  |  |  |  |
| Smoker during pregnancy | 459 | *22.5* | 65 | *23.3* |  | 66,915 | *14.8* | 1130 | *21.1* |  |
| Chronic condition (asthma, diabetes, hypertension) | 296 | *14.5* | 29 | *10.4* |  | 53,172 | *11.8* | 493 | *9.2* |  |
| Pregnancy complication (gestational diabetes, preeclampsia, placenta praevia) | 309 | *15.1* | 22 | *7.9* |  | 42,149 | *9.3* | 175 | *3.3* |  |
| Previous caesarean section | 379 | *18.6* | 19 | *6.8* |  | 73,301 | *16.2* | 244 | *4.5* |  |
| Postpartum haemorrhage>500mls | 291 | *14.3* | <10 | *<3.6* |  | 49,815 | *11.0* | 222 | *4.1* |  |
| Live birth discharged home on day of birth | Not applicable | | | |  | 10,963 | *2.4* | 4002 | *74.5* |  |

^a^ Exact numbers are not shown for small cells to maintain confidentiality. To prevent calculation of these numbers, exact numbers are also not shown for the 2^nd^ least prevalent group.

Table S4. The risk of severe maternal morbidity (SMM), transfusion and curettage with general anaesthetic during the birth of a singleton stillbirth compared to livebirths in Western Australia (2000-2015), by gestational age group.

|  | Stillbirth  n=2319 | | Livebirth  n= 456,320 |  |  |
| --- | --- | --- | --- | --- | --- |
|  | n (row %) | Total (column %) | N (row %) | Total (column %) | Adjusted RR (95% CI) |
| SMM |  |  |  |  |  |
| Gestational age (completed weeks) |  |  |  |  |  |
| 20-23 | 89 (11.2) | 793 (34.2) | 29 (11.4) | 254 (0.1) | 0.97 (0.67, 1.42) |
| 24-27 | 22 (7.3) | 301 (13.0) | 86 (7.7) | 1116 (0.2) | 0.96 (0.61, 1.51) |
| 28-31 | 25 (9.8) | 256 (11.0) | 166 (6.8) | 2433 (0.5) | 1.40 (0.94, 2.09) |
| 32-36 | 37 (9.8) | 376 (16.2) | 1069 (3.9) | 27381 (6.0) | 2.50 (1.83, 3.42) |
| ≥37 | 34 (6.1) | 556 (24.0) | 8269 (2.0) | 423244 (92.8) | 2.94 (2.12, 4.08) |
| Missing |  | 37 (1.6) |  | 1892 (0.4) |  |
| Transfusion |  |  |  |  |  |
| Gestational age (completed weeks) |  |  |  |  |  |
| 20-23 | 40 (5.0) | 793 (34.2) | 18 (7.1) | 254 (0.1) | 0.71 (0.42, 1.21) |
| 24-27 | 14 (4.7) | 301 (13.0) | 54 (4.8) | 1116 (0.2) | 0.98 (0.55, 1.74) |
| 28-31 | 18 (7.0) | 256 (11.0) | 91 (3.7) | 2433 (0.5) | 1.81 (1.11, 2.97) |
| 32-36 | 27 (7.2) | 376 (16.2) | 488 (1.8) | 27381 (6.0) | 3.95 (2.72, 5.73) |
| ≥37 | 25 (4.5) | 556 (24.0) | 3823 (0.9) | 423244 (92.8) | 4.53 (3.09, 6.65) |
| Curettage with general anaesthetic |  |  |  |  |  |
| Gestational age (completed weeks) |  |  |  |  |  |
| 20-23 | 39 (4.9) | 793 (34.2) | 10 (3.9) | 254 (0.1) |  |
| 24-27 | <10 (<3.3) | 301 (13.0) | <10 (<0.9) | 1116 (0.2) |  |
| 28-31 | <10 (<3.9) | 256 (11.0) | 11 (0.5) | 2433 (0.5) |  |
| 32-36 | 0 | 376 (16.2) | 59 (0.2) | 27381 (6.0) |  |
| ≥37 | <10 (<1.8) | 556 (24.0) | 634 (0.1) | 423244 (92.8) |  |

CI confidence interval; RR relative risk; SMM severe maternal morbidity

^a^ Adjusted for maternal ethnic origin.

Table S5. The risk of severe maternal morbidity (SMM) during the birth of a singleton stillbirth compared to livebirths in Western Australia (2000-2015), stratified by maternal comorbidities, limited to one birth per mother.^a^

|  | Without maternal comorbidities^b^ | | |  | With maternal comorbidities^b^ | | |  | |  | |
| --- | --- | --- | --- | --- | --- | --- | --- | --- | --- | --- | --- |
|  | Stillbirth  n=1224 | Livebirth  n= 160,029 |  |  | Stillbirth  n=1054 | Livebirth  109,173 |  | |  | |  |
|  | n (%) | n (%) | Adjusted^c^ RR (95% CI) |  | n (%) | n (%) | Adjusted^c^ RR (95% CI) | | *Interaction P value* ^d^ | |  |
| SMM composite | 54 (4.4) | 2852 (1.8) | 2.23 (1.71, 2.91) |  | 152 (14.4) | 2949 (2.7) | 4.85 (4.16, 5.66) | | <0.001 | |  |
| SMM composite without transfusion | 40 (3.3) | 1666 (1.0) | 2.94 (2.15, 4.01) |  | 74 (7.0) | 1639 (1.5) | 4.36 (3.47, 5.48) | | 0.055 | |  |
| SMM composite without curettage with general anaesthetic | 31 (2.5) | 2645 (1.7) | 1.37 (0.97, 1.95) |  | 128 (12.1) | 2835 (2.6) | 4.25 (3.58, 5.04) | | <0.001 | |  |
| SMM composite without transfusion and curettage with general anaesthetic | 15 (1.2) | 1420 (0.9) | 1.29 (0.78, 2.15) |  | 49 (4.6) | 1500 (1.4) | 3.16 (2.38, 4.20) | | 0.003 | |  |
|  |  |  |  |  |  |  |  | |  | |  |
| Individual procedures^e^ |  |  |  |  |  |  |  | |  | |  |
| Transfusion | 19 (1.6) | 1281 (0.8) | 1.66 (1.06, 2.60) |  | 104 (9.9) | 1457 (1.3) | 6.45 (5.30, 7.86) | | <0.001 | |  |
| Curettage with general anaesthetic | 26 (2.1) | 252 (0.2) | 12.75 (8.48, 19.16) |  | 25 (2.4) | 142 (0.1) | 16.30 (10.71, 24.79) | | 0.375 | |  |
| Hysterectomy | <10 | 19 (0.0) |  |  | 13 (1.2) | 137 (0.1) | 8.84 (4.80, 16.27) | |  | |  |
| Ventilation | <10 | 24 (0.0) |  |  | 11 (1.0) | 55 (0.1) | 18.34 (9.41, 35.76) | |  | |  |
|  |  |  |  |  |  |  |  | |  | |  |
| Individual diagnoses^c^ |  |  |  |  |  |  |  | |  | |  |
| Uterine rupture | <10 | 36 (0.0) |  |  | 10 (0.9) | 121 (0.1) | 8.72 (4.56, 16.68) | |  | |  |

CI confidence interval; RR relative risk; SMM severe maternal morbidity

^a^ One birth per mother was randomly chosen using the maternal identifier.

^b^ See Table S1 for full list of factors in the maternal comorbidities composite.

^c^ Adjusted for maternal ethnic origin and method of birth.

^d^ The interaction P value was obtained by including an interaction term for stillbirth and maternal comorbidities in the model unstratified by maternal comorbidity.

^e^See Table S2 for full list of factors in the severe maternal morbidity composite. for Individual factors only tabulated and RRs estimated where at least 10 women in the stillbirth group had this item.

Table S6. The risk of severe maternal morbidity (SMM) during the birth of a singleton stillbirth compared to livebirths in Western Australia (2000-2015), stratified by maternal comorbidities, (based on HMDC data for everything except previous caesarean section)

|  | Without maternal comorbidities^a^ | | |  | With maternal comorbidities^a^ | | |  | |  | |
| --- | --- | --- | --- | --- | --- | --- | --- | --- | --- | --- | --- |
|  | Stillbirth  n=1527 | Livebirth  n= 319,016 |  |  | Stillbirth  n=792 | Livebirth  137,304 |  | |  | |  |
|  | n (%) | n (%) | Adjusted^b^ RR (95% CI) |  | n (%) | n (%) | Adjusted^b^ RR (95% CI) | | *Interaction P value* ^c^ | |  |
| SMM composite | 73 (4.8) | 5567 (1.7) | 2.52 (2.00, 3.16) |  | 136 (17.2) | 4096 (3.0) | 5.08 (4.32, 5.98) | | <0.001 | |  |
| SMM composite without transfusion | 53 (3.5) | 3254 (1.0) | 3.22 (2.45, 4.23) |  | 63 (8.0) | 2313 (1.7) | 4.33 (3.38, 5.54) | | 0.066 | |  |
| SMM composite without curettage with general anaesthetic | 43 (2.8) | 5133 (1.6) | 1.60 (1.19, 2.16) |  | 117 (14.8) | 3948 (2.9) | 4.53(3.79, 5.41) | | <0.001 | |  |
| SMM composite without transfusion and curettage with general anaesthetic | 20 (1.3) | 2724 (0.9) | 1.46 (0.94, 2.26) |  | 44 (5.6) | 2137 (1.6) | 3.53 (2.63, 4.74) | | 0.006 | |  |
|  |  |  |  |  |  |  |  | |  | |  |
| Individual procedures^d^ |  |  |  |  |  |  |  | |  | |  |
| Transfusion | 30 (2.0) | 2508 (0.8) | 2.21 (1.55, 3.16) |  | 94 (11.9) | 1995 (1.5) | 6.82 (5.54, 8.39) | | <0.001 | |  |
| Curettage with general anaesthetic | 34 (2.1) | 544 (0.2) | 12.28 (8.62, 17.51) |  | 19 (2.4) | 181 (0.1) | 15.46 (9.62, 24.83) | | 0.236 | |  |
| Hysterectomy | <10 | 33 (0.0) |  |  | 11 (1.4) | 241 (0.2) | 7.17 (3.79, 13.57) | |  | |  |
| Ventilation | <10 | 38 (0.0) |  |  | 11 (1.4) | 73 (0.1) | 23.51 (12.32, 44.87) | |  | |  |

CI confidence interval; HMDC Hospital Morbidity Data Collection; RR relative risk; SMM severe maternal morbidity

^a^ See Table S1 for full list of factors in the maternal comorbidities composite.

^b^ Adjusted for maternal ethnic origin.

^c^ The interaction P value was obtained by including an interaction term for stillbirth and maternal comorbidities in the model unstratified by maternal comorbidity. ^d^ See Table S2 for full list of factors in the severe maternal morbidity composite. for Individual factors only tabulated and RRs estimated where at least 10 women in the stillbirth group had this item

Table S7. The risk of severe maternal morbidity (SMM) during the birth of a singleton stillbirth compared to livebirths in Western Australia (2000-2015), among those with maternal comorbidities^a^, excluding placental abruption.

|  |  | Stillbirth  n=862 | Livebirth  n= 183,928 |  |
| --- | --- | --- | --- | --- |
|  |  | n (%) | n (%) | Adjusted^b^ RR (95% CI) |
| SMM composite |  | 76 (8.8) | 4758 (2.6) | 3.04 (2.43, 3.80) |
| SMM composite without transfusion |  | 53 (6.1) | 2762 (1.5) | 3.80 (2.90, 4.98) |
| SMM composite without curettage with general anaesthetic |  | 51 (5.9) | 4552 (2.5) | 2.13 (1.62, 2.81) |
| SMM composite without transfusion and curettage with general anaesthetic |  | 28 (3.2) | 2512 (1.4) | 2.22 (1.53, 3.22) |
|  |  |  |  |  |
| Individual procedures^c^ |  |  |  |  |
| Transfusion |  | 34 (3.9) | 2220 (1.2) | 2.79 (1.99, 3.93) |
| Curettage with general anaesthetic |  | 25 (2.9) | 256 (0.1) | 18.35 (12.02, 28.01) |

CI confidence interval; RR relative risk; SMM severe maternal morbidity

^a^ See Table S1 for full list of factors in the maternal comorbidities composite.

^b^ Adjusted for maternal ethnic origin.

^c^ See Table S2 for full list of factors in the severe maternal morbidity composite. for Individual factors only tabulated and RRs estimated where at least 10 women in the stillbirth group had this item.

.

Table S8. Maternal and other factors associated with multiple pregnancies at 20 or more weeks’ gestation in Western Australia (2000- 2015).

|  | Only livebirths | | Stillbirth(s) and livebirth(s) | | Only stillbirths | | Any stillbirth | |
| --- | --- | --- | --- | --- | --- | --- | --- | --- |
|  | n=6936 | | n=113 | | n=81 | | n=194 | |
|  | n | *%* | n | *%* | n | % | n | *%* |
| Maternal age (years) |  |  |  |  |  |  |  |  |
| <25 | 888 | *13.2* | 12 | *10.6* | 13 | *16.0* | 25 | *12.9* |
| 25-29 | 1708 | *25.4* | 25 | *22.1* | 20 | *24.7* | 45 | *23.2* |
| 30-34 | 2296 | *34.1* | 41 | *36.3* | 28 | *34.6* | 69 | *35.6* |
| >34 | 1844 | *27.4* | 35 | *31.0* | 20 | *24.7* | 55 | *28.4* |
|  |  |  |  |  |  |  |  |  |
| Parity |  |  |  |  |  |  |  |  |
| 0 | 2809 | *41.7* | 50 | *44.2* | 34 | *42.0* | 84 | *43.3* |
| 1 | 2224 | *33.0* | 37 | *32.7* | 32 | *39.5* | 69 | *35.6* |
| ≥2 | 1703 | *25.3* | 26 | *23.0* | 15 | *18.5* | 41 | *21.1* |
|  |  |  |  |  |  |  |  |  |
| Plurality |  |  |  |  |  |  |  |  |
| 2 | 6624 | *98.3* |  |  |  |  |  |  |
| >2 | 112 | *1.7* | <10 ^a^ |  | <10 ^a^ |  | <10 ^a^ |  |
|  |  |  |  |  |  |  |  |  |
| Maternal ethnic origin |  |  |  |  |  |  |  |  |
| Caucasian | 5477 | *81.3* |  |  |  |  | 148 | *76.3* |
| Aboriginal | 317 | *4.7* | <10 ^a^ |  | <10 ^a^ |  | 14 | *7.2* |
| Other | 942 | *14.0* |  |  |  |  | 32 | *16.5* |
|  |  |  |  |  |  |  |  |  |
| SES area birth residence tertiles |  |  |  |  |  |  |  |  |
| Lowest | 2119 | *31.5* | 37 | *32.7* | 31 | *38.3* | 68 | *35.1* |
| Middle | 2083 | *30.9* | 36 | *31.9* | 24 | *29.6* | 60 | *30.9* |
| Highest | 2273 | *33.7* | 38 | *33.6* | 22 | *27.2* | 60 | *30.9* |
| Missing values^b^ | 261 | *3.9* | 2 | *1.8* | 4 | *4.9* | 6 | *3.1* |
|  |  |  |  |  |  |  |  |  |
| Remote or very remote birth residence | 597 | *8.9* | <10 ^a^ |  | <10 ^a^ |  | 14 | *7.2* |
|  |  |  |  |  |  |  |  |  |
| Smoker during pregnancy | 900 | *13.4* | 15 | *13.3* | 11 | *13.6* | 26 | *13.4* |
|  |  |  |  |  |  |  |  |  |
| Gestational age (completed weeks) |  |  |  |  |  |  |  |  |
| 20-23 | 39 | *0.6* |  |  |  |  | 69 | *35.6* |
| 24-27 | 158 | *2.3* |  |  |  |  | 30 | *15.5* |
| 28-31 | 503 | *7.5* |  |  | <10 ^a^ |  | 22 | *11.3* |
| 32-36 | 3523 | *52.3* |  |  | <10 ^a^ |  | 41 | *21.1* |
| ≥37 | 2508 | *37.2* |  |  | <10 ^a^ |  | 31 | *16.0* |
|  |  |  |  |  |  |  |  |  |
| Onset of labour |  |  |  |  |  |  |  |  |
| Spontaneous | 2098 | *31.1* |  |  |  |  | 88 | *45.4* |
| Induced | 1461 | *21.7* |  |  |  |  | 45 | *33.5* |
| Prelabour caesarean section | 3177 | *47.2* |  |  | <10 ^a^ |  | 41 | *21.1* |
|  |  |  |  |  |  |  |  |  |
| Birth method |  |  |  |  |  |  |  |  |
| Only vaginal | 2249 | *33.4* |  |  |  |  | 129 | *66.5* |
| Vaginal and caesarean section | 92 | *1.4* |  |  |  |  | 0 | *0.0* |
| Only caesarean sections | 4395 | *65.2* |  |  | <10 ^a^ |  | 65 | *33.5* |
|  |  |  |  |  |  |  |  |  |
| Any comorbidity^c^ | 3452 | *51.2* | 51 | *45.1* | 41 | *50.6* | 92 | *47.4* |
| Any chronic/existing condition | 1381 | *20.5* | 30 | *26.5* | 20 | *247* | 50 | *25.8* |
| Pregnancy complications/risk factors | 2655 | *39.4* | 34 | *30.1* | 27 | *33.1* | 61 | *31.4* |

^a.^ Exact numbers and percentages are not shown for small cells to maintain confidentiality. To prevent calculation of these numbers, only the numbers for any stillbirth are given in these cases.

^b^. Missing values only given if >3% missing

c. Maternal comorbidities were chronic or pregnancy-specific conditions recorded in the Midwives Notification System or Hospital Morbidity Data Collection (see Table S1 for details).

Table S9 The risk of severe maternal morbidity (SMM) during the births of multiple pregnancies with any stillbirth compared to multiple pregnancies with only livebirths in Western Australia (2000-2015)

|  | Any stillbirth^a^  n=194 | Only livebirth(s)  n=6736 |  |
| --- | --- | --- | --- |
|  | n (%) | n (%) | Adjusted^b^ RR (95% CI) |
| SMM composite | 17 (8.8) | 257 (3.8) | 2.21 (1.72, 2.82) |
| SMM composite without transfusion | 11 (5.7) | 99 (1.5) | 3.73 (2.98, 4.67) |
| SMM composite without curettage with general anaesthetic | 10 (5.2) | 236 (3.5) | 1.41 (1.04, 1.91) |

CI confidence interval; RR relative risk; SMM severe maternal morbidity.

^a.^ Because of small numbers and similar relative risks associated with the only stillbirths and Stillbirth(s) and livebirth(s) groups, only results for any stillbirth are shown.

^b.^ Adjusted for maternal ethnic origin.


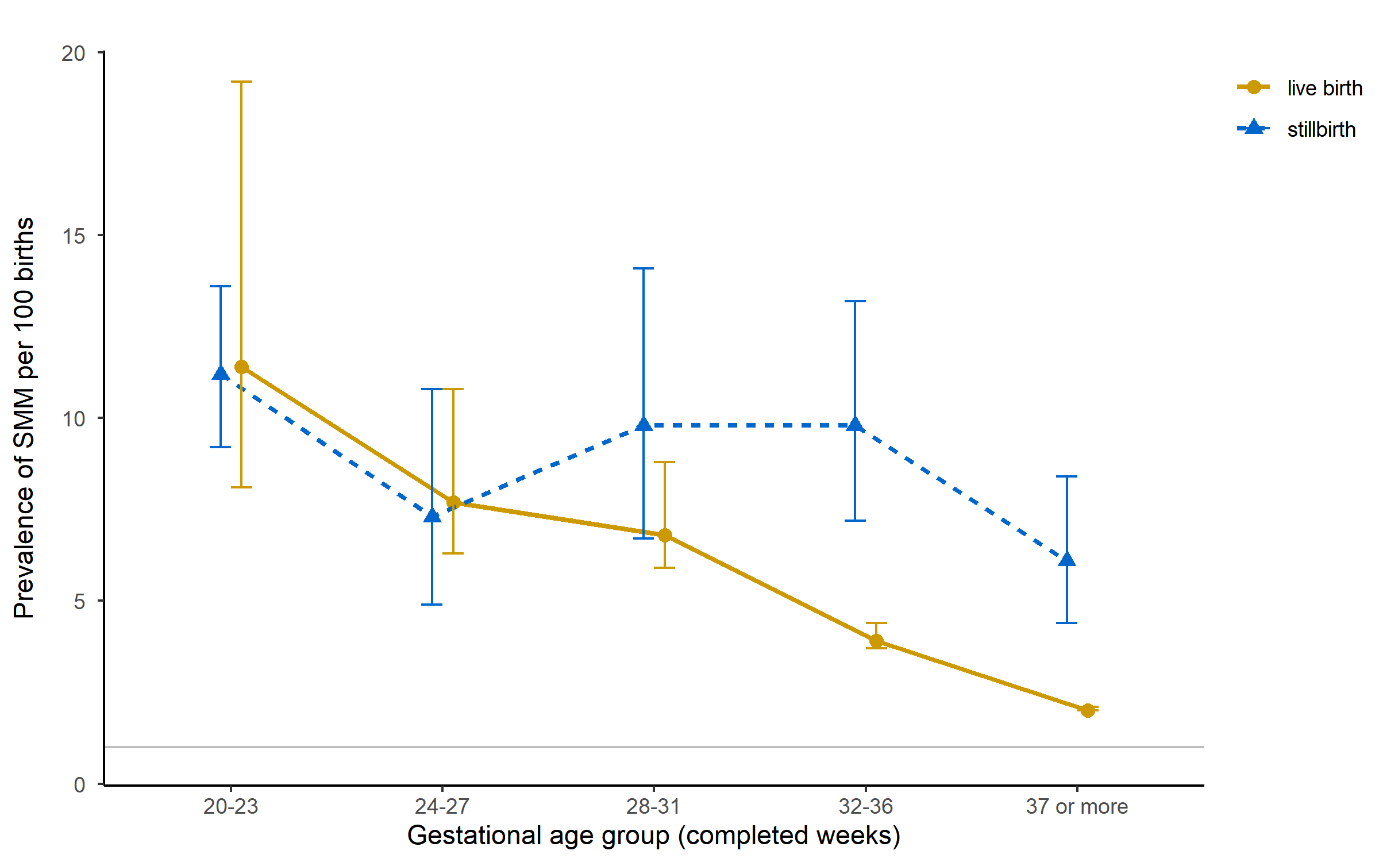


Figure S1: Rates of severe maternal morbidity by gestational age group among live births and stillbirths in Western Australia, 2000-2015.
